# Veterinary Medical Association of New York County

**Published:** 1897-03

**Authors:** Robert W. Ellis

**Affiliations:** Secretary


### VETERINARY MEDICAL ASSOCIATION OF NEW YORK COUNTY.

THE regular monthly meeting of this Association was called to order at 8.45 P.M., February 3d, at its rooms in the Academy of Medicine, with the President, Dr. Huidekoper, in the chair.

The following members responded to roll-call: Drs. Bretherton, Bell, C. C. Cattanach, J. S. Cattanach, Delaney, Ellis, Farley, Gill, Huidekoper, Hanson, Jackson, Loomis, Lamkin, Machan, MacKellar, Miller, Neher, and O'Shea. (18.)

Dr. Gill, Chairman of the Board of Censors, reported that the committee recommended to the Association that the action previously taken in the Glover case stand. The committee reported favorably on the name of Dr. M. J. Murphy, Jr., who had applied for membership in the Association in regular form. The committee also reported favorably on the recommendation for honorary membership of the name of Prof. A. Liautard, by members O'Shea, Robertson, and Hanson. It was then regularly moved and seconded that the report be acted upon in section; carried.

Section 1, Glover Case. Moved and seconded that the report be accepted; carried. Section 2, application of Dr. Murphy for membership. Moved and seconded that the report be accepted; carried. Section 3, recommendation of Prof. Liautard for honorary membership. The chair, after appointing a chairman *pro tem.*, offered the following protest against the admission of Prof. Liautard to honorary membership: "Mr. Chairman: It is with extreme regret that I cannot express my personal feeling of friendship for Dr. Liautard by seconding his proposal for honorary membership, but am forced to protest against it. This I must do as a matter of principle, upon the same grounds that I once prevented the election of Prof. James Law, of Cornell, as an honorary member of the U. S. V. M. A. As a leader of the profession, and on account of Prof. Liautard's long eminent standing in it, he should have been a leader in our Association; on the contrary, when we were struggling for existence he saw fit to resign, and the wording of his resignation was a grave reflection upon the Association. Now we have struggled and made a success of the Association, and I move that the proposition for honorary membership be not accepted." Seconded by Dr. Gill, who stated that he did not believe Dr. Liautard, if he knew it, would have permitted the application to have been made; carried.

Dr. Neher reported a case of "Swelling" of the face, with inflammation in the buccal cavity, followed by sloughing to the extent of exposing a portion of the supermaxillary bone and a portion of the palatine artery, and asked what might be the cause; he attributed it to "purpura." An extremely interesting discussion followed as to the etiology, etc., which was participated in by Drs. C. C. Cattanach, Miller, Farley, J. S. Cattanach, Hanson, and others. Prof. Bell expressed his belief that it was of traumatic origin, citing a case in which a similar result was brought about by a piece of hard wood becoming lodged crosswise between the third molars on either side. President Huidekoper believed it to be purpura, and gave a very interesting little "talk" in relation to it and upon the minute anatomy of the parts.

Dr. O'Shea, Chairman of the Judiciary Committee, called the attention of the meeting to Assembly Bill No. 231, introduced by Mr. Hughes, in relation to the appointment of a State Veterinarian as a member of the State Board of Health. Moved by Dr. Hanson that it be the sense of the meeting to do all they can to oppose the bill; seconded; carried.

Assembly Bills Nos. 25 and 137 were also mentioned, which, of course, had already been opposed by most of the members present. The committee again promised to take action in reference to the jury bill. Moved and seconded that the proper means be taken to have Mr. Mulvey's name removed from the register; carried. Moved and seconded that the Judiciary Committee take action in regard to the same; carried.

President Huidekoper reported for the Publication Committee that they have verified almost the entire list for Greater New York, and expect to have the work in publication by the end of February. Moved and seconded that the report be accepted; carried.

The resignation of Thomas Giffen was next taken up. It was moved and seconded that the resignation be accepted; carried.

Moved and seconded that the meeting adjourn; carried.

Robert W. Ellis, D.V.S.,
Secretary.

